# Identification of regulatory networks and hub genes controlling soybean seed set and size using RNA sequencing analysis

**DOI:** 10.1093/jxb/erw460

**Published:** 2017-01-13

**Authors:** Juan Du, Shoudong Wang, Cunman He, Bin Zhou, Yong-Ling Ruan, Huixia Shou

**Affiliations:** 1 State Key Laboratory of Plant Physiology and Biochemistry, College of Life Sciences, Zhejiang University, 866 Yuhangtang Road, Hangzhou, China; 2 Institute of Crop Science, Anhui Academy of Agricultural Sciences, Hefei, China; 3 School of Environmental and Life Sciences, The University of Newcastle, Callaghan, NSW, Australia

**Keywords:** Seed development, seed number, seed size, soybean, transcriptomic analysis

## Abstract

To understand the gene expression networks controlling soybean seed set and size, transcriptome analyses were performed in three early seed developmental stages, using two genotypes with contrasting seed size. The two-dimensional data set provides a comprehensive and systems-level view on dynamic gene expression networks underpinning soybean seed set and subsequent development. Using pairwise comparisons and weighted gene coexpression network analyses, we identified modules of coexpressed genes and hub genes for each module. Of particular importance are the discoveries of specific modules for the large seed size variety and for seed developmental stages. A large number of candidate regulators for seed size, including those involved in hormonal signaling pathways and transcription factors, were transiently and specifically induced in the early developmental stages. The soybean homologs of a brassinosteroid signaling receptor kinase, a brassinosteroid-signaling kinase, were identified as hub genes operating in the seed coat network in the early seed maturation stage. Overexpression of a candidate seed size regulatory gene, *GmCYP78A5*, in transgenic soybean resulted in increased seed size and seed weight. Together, these analyses identified a large number of potential key regulators controlling soybean seed set, seed size, and, consequently, yield potential, thereby providing new insights into the molecular networks underlying soybean seed development.

## Teaser

Comparative RNA sequencing analyses on two soybean genotypes with contrasting seed size across seed set, growth, and early maturation stages identified key genes, modules, and regulatory networks controlling seed development.

## Introduction

Soybean [*Glycine max* (L.) Merr.] is the most widely grown grain legume in the world. It provides an important source of protein and oil (Ainsworth *et al.*, 2012). As for other major crops, grain yield is always the most important trait in soybean breeding and cultivation. The first whole-genome sequence of the variety Williams 82, completed in 2010, provides a powerful resource for soybean functional genomic research (Schmutz *et al.*, 2010). Genome-scale technologies, such as microarrays and cDNA sequencing, have been employed to isolate genes and reveal regulatory networks. These genomic approaches can be used to identify and manipulate key genetic bottlenecks that limit soybean seed set and size for high-yield breeding.

Compared with Arabidopsis and rice, only limited transcriptome data are reported on seed development in soybean (Asakura *et al.*, 2012; Jones and Vodkin, 2013; Lu *et al.*, 2016; Sha *et al.*, 2012). In this context, Jones *et al.* (2010) identified a number of seed-specific genes in soybean using microarray chips consisting of 27000 cDNAs (Vodkin *et al.*, 2004). They found that the expression levels of genes related to cell growth and cellular maintenance, as well as photosynthesis, decreased as the cotyledons approached the mature stage, whereas genes encoding storage proteins had their highest expression levels at the stage of highest fresh weight. Interestingly, some transcription factor genes were expressed at higher levels in the desiccating stage than in most of the green stages (Jones *et al.*, 2010). The same authors conducted gene expression profiling at seven different stages of soybean seed development, spanning the period from a few days post-fertilization to the mature seed, using RNA sequencing (RNA-Seq) in the cultivar Williams (Jones and Vodkin, 2013). Recently, a transcriptome analysis was performed during early and middle seed maturation stages using 8 cultivated and 10 wild soybean varieties (Lu *et al.*, 2016). The study identified 2,680 genes that are differentially expressed in these maturation stages. Two cultivar-specific gene coexpression networks were established. The key regulators identified in the study, *GA20OX* and *NFYA*, were found to increase seed size and seed oil content in transgenic Arabidopsis (Lu *et al.*, 2016). While these transcriptome analyses provided the first sets of expression data on genes controlling the mid-to-mature stage of seed development, there is a lack of knowledge to enable a comprehensive understanding of soybean seed size control in the early developmental stage of seed set and growth.

Seed yield is the product of seed number and seed size, factors that are largely determined at early seed developmental stages (Ruan *et al.*, 2012) and coordinately controlled by maternal and filial tissues (Weber *et al.*, 2005). From fertilization to seed maturity, soybean seed development can be divided into three stages: pre-embryo (Soybean Ontology: Soyb: 0001285-0001289; Grant *et al.*, 2010), embryo growth (Soybean Ontology: Soyb: 0001290), and seed maturity and desiccation stages (Soybean Ontology: Soyb: 0001291-0001294) (see Supplementary Table S1 at *JXB* online). These stages correspond to the three major seed developmental phases of seed set, seed growth, and seed maturation (Weber *et al.*, 2005; Ruan *et al.*, 2012). Seed set refers to the transition from ovule to seed upon fertilization and is characterized by extensive cell division and coordinated development of maternal and filial tissues (Ruan *et al.*, 2012; Supplementary Table S1). The seed growth stage features cell expansion and synthesis of storage products in the newly formed embryo or endosperm. At this stage, the cotyledons (the main parts of the embryo) have formed and begin to undergo cell expansion. The seed set stage determines seed yield potential through the establishment of the number of seeds and, to a large degree, their final size through controlling seed cell number by regulating cell division. The seed growth and early seed maturation stages determine the final seed size and weight through the accumulation of storage products (Weber *et al.*, 2005).

Seed size is determined by coordinated development of the maternal seed coat and the filial endosperm and embryo (Garcia *et al.*, 2005; Ohto *et al.*, 2009). The seed coat serves as a transient storage organ in the early stage of seed development, which accumulates starch and proteins before storage activity starts in the embryo of most grain legumes (Déjardin *et al.*, 1997). The seed coat also contains the vascular bundles of the seed where photoassimilates and water are unloaded as essential resources to support the growth of the filial tissues (Ruan *et al.*, 1997). Phloem-unloaded sucrose, the major photoassimilate, needs to be degraded in the seed coat by invertases into hexoses, which serve as critical nutrients and signals to support filial tissue development (Wang and Ruan 2016; Weber *et al.*, 1996). Final seed size is also controlled by the endosperm (Wang *et al.*, 1993). Premature endosperm cellularization causes the formation of small seeds, while delayed endosperm cellularization results in enlarged seeds (Berger *et al.*, 2006; Garcia *et al.*, 2005).

Complex signaling pathways and regulatory networks, which include sugar and hormonal signaling, transcription factors, and metabolic pathways, have been reported to be involved in the seed development of Arabidopsis (Le *et al.*, 2010; Orozco-Arroyo *et al.*, 2015; Ruan, 2014; Ruan *et al.*, 2012; Weber *et al.*, 2005). However, gene expression profiling and identification of regulatory networks that control early developmental stages in the model legume crop soybean are yet to be achieved. In this study, we conducted a detailed RNA-Seq analysis for the set, growth, and early maturation stages of developing seeds in two soybean genotypes with contrasting seed size phenotypes. The data set for the early seed developmental stages provides a comprehensive and systems-level view on the dynamic gene expression networks and their potential roles in controlling seed size. Using pairwise comparisons and weighted gene coexpression network analysis (WGCNA), we identified modules of coexpressed genes and candidate hub genes for each developmental stage and for genotypes with different seed size. We further overexpressed a candidate seed size regulatory gene, *GmCYP78A5*, in soybean. The transgenic soybean lines exhibited enlarged seed size and increased seed weight, as predicted. This work provides important insights into the molecular networks underlying soybean seed development.

## Materials and methods

### Plant growth and RNA sample collection

Two soybean varieties with contrasting seed size were used in this study. The large seed variety Wandou 28 (V1) is an elite variety originating from the Anhui province of China, whereas the small seed variety Peixian Layanghuang (V2) is a landrace germplasm from the Jiangsu province of China (Chinese Crop Germplasm Information System, accession number ZDD03969; http://icgr.caas.net.cn/cgris_english.html). The average dry weight of 100 seeds was 28.11 g for V1 and 7.24 g for V2. V1 and V2 were planted in the greenhouse of the agricultural station of Zhejiang University in Hangzhou, China, in 2015. The sampling points of seed set (S1), seed growth (S2), and early seed maturation (S3) were at 5–7, 10–14, and 20–24 days after fertilization (Table 1 and Supplementary Table S1). After sampling, the tissues were quickly frozen in liquid nitrogen and stored at –70^o^C until RNA isolation. Three biological replicates were used for each of the sampling points.

**Table 1.** Sample description and numbers of RNA-Seq reads for samples

Developmental stage		Sampled tissues	Genotype^*^	Number of reads**		
				Rep 1	Rep 2	Rep 3
S1 3–5 DAF ^***^	**Seed set:** Acellular endosperm in seed, undergoing active cell division in embryo and maternal seed coat and seed pod.	Seed pod with whole seed	V1	77.1 M	64.6 M	63.1 M
			V2			
S2 10–14 DAF	**Seed growth:** Endosperm is degenerated and its cellular contents are assimilated by the cotyledons.	Whole seed	V1	66.4 M	64.6 M	76.2 M
			V2	63.3 M	68.2 M	65.2 M
S3-1 20–22 DAF	**Seed filling:** Seed coat and cotyledon are separated from whole seed with 90–100mg fresh weight (around one-third of final weight); accumulation of nutrients, oil, storage proteins in cotyledon.	Seed coat	V1	73.1 M	71.3 M	77.1 M
			V2	74.3 M	65.7 M	85.6 M
S3-2 20–22 DAF		Seed cotyledon	V1	70.5 M	69.4 M	74.2 M
			V2	60.3 M	61.9 M	71.4 M

* Genotype V1 and V2 are the large and small seed cultivar, respectively; ** number of processed clean reads obtained from three biological replicates of RNA high-throughput sequencing [expressed in millions (M)]. DAF, days after fertilization.

### Microscopic analyses

Six S1 stage seeds were collected and fixed for 12 h at 4 °C in 2% glutaraldehyde in 0.02 M phosphate buffer at pH 7.0. Samples were dehydrated in an ethanol series and acetone, and embedded in Spurr’s resin (SPI-CHEM). Sections 5µm thick were cut with a rotary microtome (Leica) and stained with Toluidine blue O for examination under a Zeiss microscope. Images were photographed using the associated Zeiss Axio Imager A1 system.

### RNA isolation, cDNA library construction, Illumina deep sequencing, and quantitative reverse transcription-PCR analysis

Total RNA was extracted using Trizol reagent (Invitrogen, CA, USA) following the manufacturer’s protocol. RNA integrity was confirmed by using the 2100 Bioanalyzer. A total of 0.5–2 μg RNA per sample was sent for library preparation using the TruSeq RNA sample preparation kit (Illumina RS-122–2101, Illumina, CA, USA). The library was sequenced on an Illumina HiSeq2000 instrument. Approximately 60–77 million 100 bp pair-end reads were generated for each sample (Table 1). Quantitative reverse transcription (RT)-PCR (qRT-PCR) analysis was performed using a LightCycler 480 machine (Roche Diagnostics). The relative expression of each gene was calculated after being normalized to the *CYCLOPHILIN2* (*CYP2*) gene. All primers for qRT-PCR are listed in Supplementary Table S14.

### Data processing of RNA-Seq experiments

Raw data in the fastq format were first processed using the NGS QC Toolkit (Patel and Jain, 2012). Clean data were obtained by removing reads containing adapter, poly-N and low-quality reads from raw data. All the downstream analyses were based on clean data with high quality determined by Q30. Differential gene and transcript expression analysis of RNA-Seq experiments were carried out using TopHat and Cufflinks. The number of total reads and reads that can be uniquely mapped to the genome are shown in Supplementary Table S17. The fragments per kilobase of transcript per million mapped reads (FPKM) and transcript level per million (TPM) count values were calculated using eXpress (Mortazavi *et al.*, 2008; see Supplementary Tables S15 and S16). Principal component analysis was performed using the DESeq (2012) R package. Scripts for the bioinformatics analysis are shown in Supplementary Table S18.

### Differential expression, cluster analysis and gene ontology enrichment analysis

Differential expression analysis was performed using the DESeq (2012) R package. Hierarchical cluster analysis was used to identify differentially expressed genes (DEGs). DEGs were filtered with expression levels FPKM >5, false discovery rate (FDR) <0.01, log_2_ fold change >1 or <–1 in each pairwise comparison. Gene ontology enrichment analysis of the DEGs was performed using the DESeq (2012) R package based on hypergeometric distribution. WGCNA was performed according to Langfelder and Horvath (2008).

### Generation of transgenic soybean plants overexpressing *GmCYP78A5*

The coding DNA sequence (CDS) of *GmCYP78A5* (*Glyma. 05G019200*) was amplified from Williams 82 developing seed (S3) cDNA. A 2kb DNA sequence of a soybean seed-specific promoter from the β-conglycinin α subunit encoding gene *Glyma.20G148300.1* (Yoshino *et al.*, 2006) was amplified from Williams 82 genomic DNA. The ligated promoter-cDNA fragment was cloned into a pTF101.1 vector (Paz *et al.*, 2004). The resulting construct (Supplementary Fig. S2) was transfected into *Agrobacterium tumefaciens* strain EHA101 to transform soybean using the cotyledonary node transformation method (Song *et al.*, 2013). Individual transgenic plants were selected through a shoot induction, shoot elongation, and root elongation process on medium containing 5 mg l^–1^ glufosinate, then further screened by detection of the presence or absence of the transgene using PCR. The primers used are listed in Supplementary Table S14.

## Results

### Morphological analysis of two soybean genotypes with contrasting seed size

**Fig. 1.**
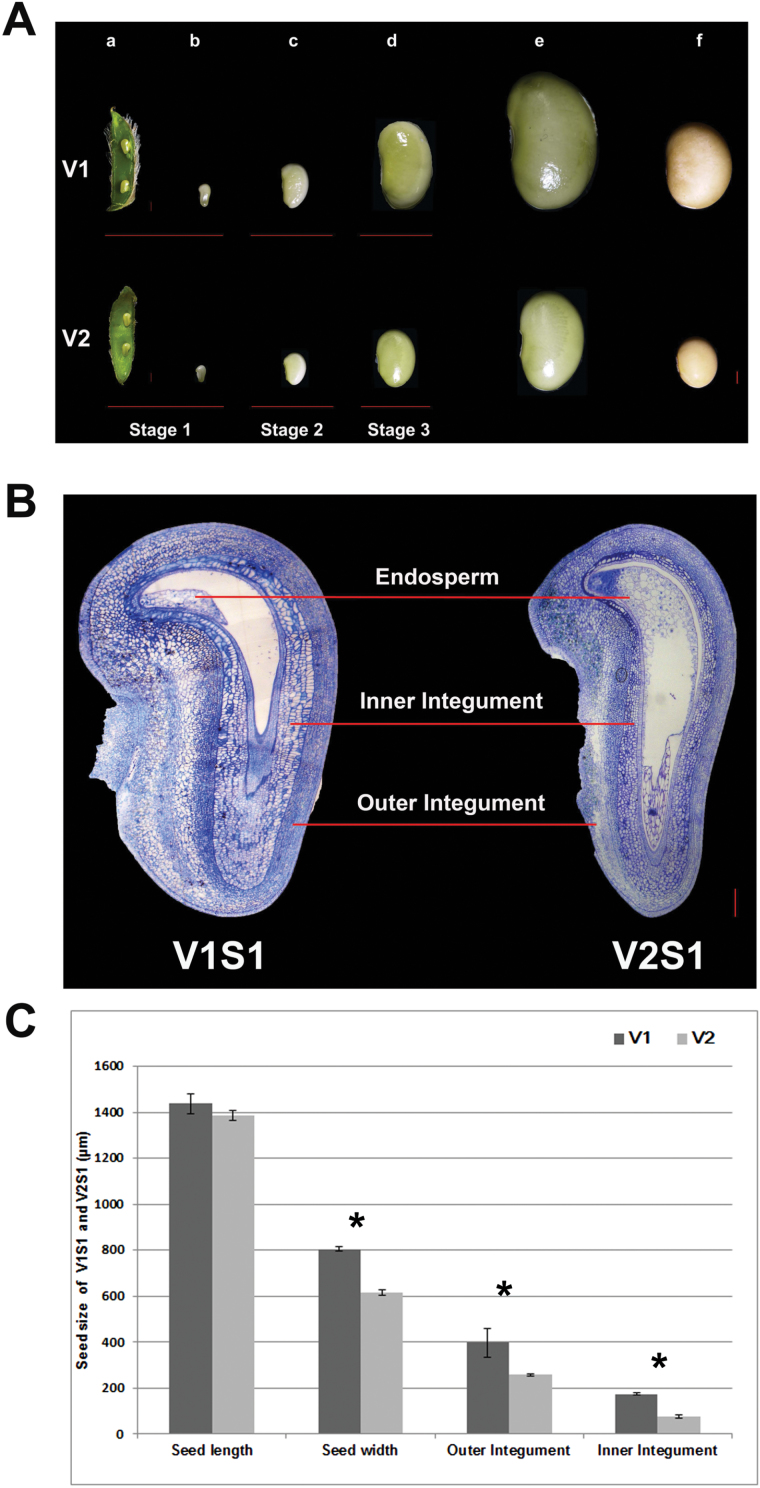
**Stages of soybean seed development and sampling times of two soybean varieties.** (A) Seed developmental stages in soybean varieties V1 and V2. Pod (a) and seed (b) at 5–7 days after fertilization (DAF) (Stage S1), seed at 10–14 DAF (Stage S2; c), seeds at 20–24 DAF (Stage S3; d), seed at maturation stage (e) and desiccation stage (f). Bars=1 mm. (B) Cross-section of the seeds of V1 and V2 at stage S1. V1 showed enlarged seed size partly due to increased cell size in the outer and inner integument compared with V2. Bars=200μm. (C) Seed lengths and widths, and widths of the outer and inner integument of V1 and V2 at stage S1. Values are presented as mean±SE. Three biological replicates were used for measurements. ** Significant difference (*P<*0.01), Student’s t-test. (This figure is available in colour at *JXB* online.)

Key developmental features of seeds at each of the sampling stages are described in Table 1. During the S1 stage, the seeds were around 0.8–1 mm in length, and surrounded and protected by the maternal tissues (Fig. 1A). Histological analysis showed that S1 seeds of V1 and V2 were both differentiated into cellular endosperm, heart stage embryo, and thickened inner and outer integuments (Fig.1B). At the S2 stage, the seed endosperm had degenerated and its cellular content had been assimilated by the cotyledons. At this stage, the cotyledons had emerged and filled all the space within the seed coat. The whole seed, however, was still thin and flat, ~2–3 mm in length at this stage (Fig. 1A). At the S3 stage, the cotyledons were undergoing filling with storage products (Ruan *et al.*, 2012). This stage is characterized by significant increases in seed size and storage products (Carlson and Lersten, 2004).

The difference in seed size between V1 and V2 appeared at the S1 stage and became more evident at stages S2 and S3 (Fig 1A, B). In stage S3, V1 showed a 61% increase in seed length and an 82% increase in seed weight compared with V2 (Supplementary Fig S1A, B). It is conceivable that the DEGs at each stage between V1 and V2 may play important roles in determining seed size; hence, these were subject to detailed investigation.

### RNA-Seq analysis on developing seeds of two soybean varieties with contrasting seed size

To explore the molecular basis of the morphological differences in seed development described above, RNA-Seq analyses were conducted to generate transcriptome profiles. Four tissues of soybean seeds at stages S1, S2, and S3 were sampled from both genotypes. These include whole pods with seeds at S1, whole seeds at S2, seed coats at S3-1, and cotyledons at S3-2. At the S1 stage, the whole pods containing seeds were sampled together, largely because of the technical difficulties in dissecting the tiny seeds from their pods without cross-contamination. The combined pod and seed sampling allowed us to obtain information about genes and regulatory networks from both maternal and filial tissues. At the S2 stage, pod walls were removed and whole seeds were sampled, as at this stage the seed coat and the filial tissues were small and tightly compacted. At the S3 stage, seed coats and cotyledons were large enough to be sampled separately. To this end, identification of the DEGs from seed coat and cotyledon could contribute to the understanding of the differential control of seed size exerted by maternal and filial tissues.

**Fig. 2.**
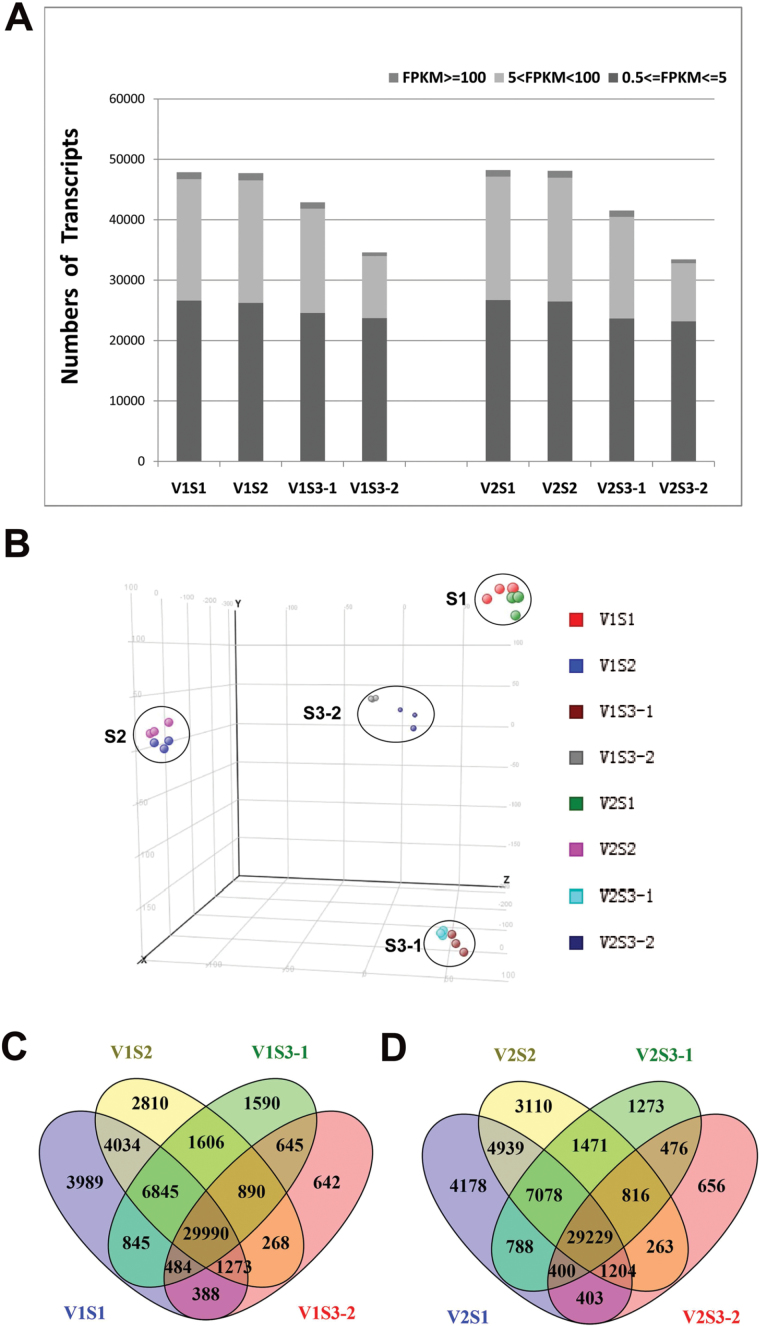
**Global gene expression profiling in early seed development stages.** (A) Numbers of detected transcripts in each sample. (B) Principal component analysis of the RNA-Seq data. (C, D) Venn diagrams of differentially expressed transcripts among the four tissues of (C) V1 and (D) V2. (This figure is available in colour at *JXB* online.)

RNA-Seq analysis was performed on the samples described above with three biological replicates for each. In total, 24 libraries were constructed and analyzed. After removing low-quality reads, the average number of reads per library was over 60 million (Table 1). The RNA-Seq reads were aligned with the reference map of the newly assembled (V2.0) soybean genome (Langmead and Salzberg, 2012; Schmutz *et al.*, 2010). The numbers of transcripts identified in each sample, expressed in FPKMs, are shown in Fig. 2A. Genes with normalized reads lower than 0.5 FPKM were removed from the analysis. In total, 47848, 47715, 42894, and 34579 transcripts were found to be expressed in S1, S2, S3-1, and S3-2 of V1, respectively. Similarly, 48218, 48109, 41530, and 33446 transcripts were identified in the samples from the respective stages of the V2 genotype. Approximately 55% of expressed genes were in the 0.5–5 FPKM range, and 40% of expressed gene were in the range 5–100 FPKM (Fig. 2A) .The RNA-Seq data were uploaded to the Sequence Read Archive of the National Center for Biotechnology Information (accession number SRR3723988; https://trace.ncbi.nlm.nih.gov/Traces/sra/?run=SRR3723988) for access by the scientific community.

qRT-PCR analysis was used to validate the quality of the RNA-Seq data. Six genes from each developmental stage were selected for qRT-PCR analysis (Supplementary Fig. S3). The strong correlation between the RNA-Seq and qRT-PCR data indicates the reliability of our transcriptomic profiling data (Supplementary Fig. S3).

Principal component analysis revealed that the eight samples could be clearly assigned to four groups as S1, S2, S3-1, and S3-2 (Fig. 2B). V1 and V2 samples from the same stage were clustered together, suggesting that the overall transcriptome profiling is similar for V1 and V2 at each developmental stage. The overlaps of expressed genes in the four samples of V1 and V2 are shown in Fig. 2C and 2D.

### Identification of differentially expressed genes between the genotypes

**Fig. 3.**
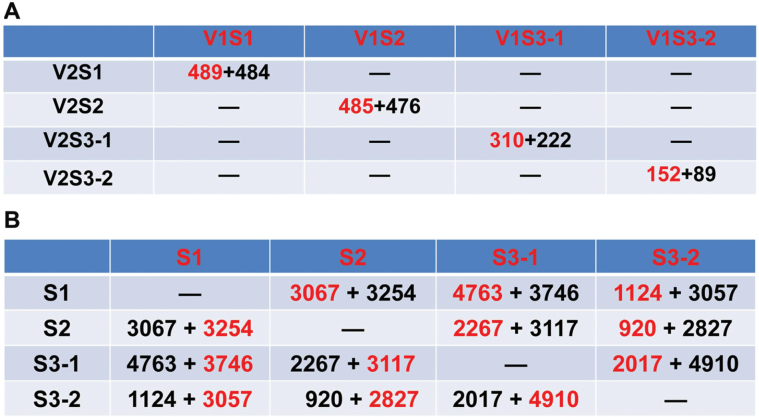
**Numbers of DEGs in each developmental stage and tissues.** (A) Numbers of DEGs between V1 and V2 at each developmental stage. (B) Numbers of DEGs in each developmental stage of V1 and V2. DEGs were filtered according to FPKM >5, FDR <0.01, log_2_ fold change >1, or log_2_ fold change <–1. Numbers in red or black indicate the number of up-regulated or down-regulated genes when the sample in red is compared with the sample in black, respectively. (This figure is available in colour at *JXB* online.)

To identify the genes correlating with the large seed genotype in the seed set, seed growth, and early seed maturation stage, we conducted pairwise comparison at each developmental stage between genotypes V1 and V2. The DEGs were filtered with expression levels FPKM >5, FDR <0.01, log_2_ fold change >1 or <–1 in each pairwise comparison (Supplementary Table S2). At the S1 stage, pairwise comparisons of V1S1 *versus* V2S1 showed that 973 genes were significantly differentially expressed (Fig. 3A). Among these, 489 genes were significantly up-regulated and 484 genes were significantly down-regulated in V1 relative to V2. The representative genes for the up- or down-regulated DEGs are listed in Tables 2 and 3, respectively, according to their functional categories. The transcripts of five transcription factor family genes were enriched in V1S1. A gene encoding auxin response factor 4 showed 16-fold increased expression in V1S1 relative to V2S2. Genes encoding indoleacetic acid-induced protein 8, a homeobox-leucine zipper family protein, a basic helix-loop-helix protein and X-BOX transcription factor exhibited over two-fold increased expression in the large seed V1 relative to the small seed V2 at S1. An auxin signaling pathway gene of the auxin-responsive GH3 gene showed the highest expression level in V1S1. Interestingly, there was no expression detected in V2 samples for a GH3. Other plant hormonal transduction pathway genes that showed over two-fold increased expression in V1S1 include those encoding serine/threonine-protein kinase SRK2 and a two-component response regulator of the ARR-A family Embryo-specific protein 3 (Table 2).

**Table 2.** FPKMs and functional categories of genes significantly up-regulated in V1 at S1

Gene name	V1S1	V2S1	Description
**Transcription factors**			
Glyma.13G328000	8.78	0.55	Auxin response factor 4
Glyma.01G019400	5.91	1.07	Indoleacetic acid-induced protein 8
Glyma.01G240100	5.93	1.81	Homeobox-leucine zipper family protein
Glyma.03G240000	15.66	6.88	Basic helix-loop-helix (bHLH) protein
Glyma.10G189300	13.22	3.36	X-BOX transcription factor related
Glyma.19G258300	8.2	0.01	Unknown DNA binding protein
Glyma.19G262000	5.99	0.36	Unknown DNA binding protein
**Plant hormone signal transduction**			
Glyma.11G038800	5.43	1.92	Serine/threonine-protein kinase SRK2
Glyma.11G155100	8.79	3.29	Two-component response regulator ARR-A family
Glyma.06G243500	26.13	0.01	Auxin-responsive GH3 gene family
Glyma.16G202000	5.15	0.76	Embryo-specific protein 3 (ATS3)
**Fatty acid metabolism**			
Glyma.13G318000	9.9	3.05	NAD(P)-linked oxidoreductase superfamily protein
Glyma.07G042900	13.41	4.88	Fatty acid biosynthetic process
**Protein kinase activity**			
Glyma.11G235300	13.55	3.48	CBL-interacting protein kinase 13
Glyma.18G021600	18.44	8.08	CBL-interacting protein kinase 23
Glyma.08G020800	18.26	8.11	Leucine-rich repeat transmembrane protein kinase family protein
Glyma.05G214300	8.63	1.38	Leucine-rich repeat transmembrane protein kinase family protein
**Flavonoid biosynthesis**			
Glyma.16G033700	163.88	58.41	Flavonol 3-O-glucosyltransferase
Glyma.16G175600	17.87	7.19	Flavonol 7-O-beta-glucosyltransferase

**Table 3.** FPKMs and functional categories of genes significantly down-regulated in V1 at S1

Gene name	V1S1	V2S1	Description
**Transcription factors**			
Glyma.06G131500	2.71	5.96	Dof-type zinc finger DNA-binding family protein
Glyma.20G011700	5.39	12.82	Duplicated homeodomain-like superfamily protein
Glyma.05G032200	32.1	79.74	Myb-like transcription factor family protein
Glyma.06G233300	5.38	13.71	PHD finger family protein
Glyma.07G126800	33.46	94.78	Zinc finger C-x8-C-x5-C-x3-H type family protein
Glyma.09G274000	6.36	13.08	WRKY DNA-binding domain
Glyma.15G091000	1.88	5.73	Auxin response factor 6
Glyma.12G022200	4.28	9.08	DHHC-type zinc finger family protein
Glyma.14G062700	4.18	8.86	GATA-type zinc finger transcription factor family protein
Glyma.17G080900	2.86	7.83	K-box region and MADS-box transcription factor family protein
Glyma.07G234200	3.1	9.5	Squamosa promoter binding protein-like 1
Glyma.06G238100	0.33	54.1	Squamosa promoter binding protein-like 8
Glyma.17G096700	21.97	46.05	Transcription factor HEX, contains HOX and HALZ domains
**Plant hormone signal transduction**			
Glyma.19G161100	6.26	17.16	Indole-3-acetic acid inducible 14/AUX/IAA family
Glyma.07G015200	4.07	8.62	Indole-3-acetic acid inducible 18/AUX/IAA family
Glyma.02G245600	64.07	155.15	Gibberellin-regulated family protein
Glyma.04G169600	54.48	115.29	Gibberellin-regulated family protein
Glyma.10G031900	44.43	95	Auxin-responsive protein IAA
Glyma.12G035100	1.1	6.6	SAUR family protein
**MAPK signaling pathway**			
Glyma.02G093200	2.19	54.01	Heat shock 70 kDa protein 1/8
**Flavone and flavonol biosynthesis**			
Glyma.06G295700	372.57	1014.74	Flavonol 3-O-methyltransferase
Glyma.12G109800	150.69	308.27	Flavonol 3-O-methyltransferase
Glyma.06G295700	8.31	21.45	Flavonol 3-O-methyltransferase
**Fatty acid metabolism**			
Glyma.02G273300	2.03	5.16	Enoyl reductase [EC:1.3.1.-]
Glyma.07G121500	17.72	42.39	Fatty acid hydroxylase superfamily
Glyma.10G042500	19.21	56.07	GDSL-like lipase/acylhydrolase superfamily protein
Glyma.06G183900	4.63	10.3	Aldehyde dehydrogenase (NAD+)
**Seed storage**			
Glyma.18G190000	3.18	24.62	Seed storage 2S albumin superfamily protein
**Cytochrome P450**			
Glyma.19G015200	0.75	7.5	Cytochrome P450 CYP2 subfamily
Glyma.01G122300	0.19	5.14	Cytochrome P450
Glyma.09G186300	3.88	16.44	Cytochrome P450 CYP2 subfamily
Glyma.01G122300	0.19	5.14	Cytochrome P450

At the S2 stage, 485 and 476 genes were significantly up- or down-regulated, respectively, in samples of the large seed genotype V1 compared with V2 (Fig. 3A). The representative genes for the up- or down-regulated DEGs in V1S2 are listed in Tables 4 and 5. More specifically, a MYB-like transcription factor showed 3.8-fold increased expression in V1 relative to V2. Similarly, a group of genes responsible for flavonoid biosynthesis, metabolism of starch, sucrose, and nitrogen, as well as a cytochrome P450 CYP2 subfamily gene, also showed increased expression in V1S2 compared with V2S2.

**Table 4.** FPKMs and functional categories of genes significantly up-regulated in V1 at S2

Gene name	V1S2	V2S2	Description
**Transcription factor**			
Glyma.12G100600	105.55	27.74	MYB-like DNA-binding protein
**Plant hormone signal transduction**			
Glyma.07G030100	21.86	10.13	Histidine-containing phosphotransfer protein
Glyma.08G212800	36.58	13.8	Histidine-containing phosphotransfer protein
**Flavonoid biosynthesis**			
Glyma.11G011500	33.88	14.62	Chalcone synthase
Glyma.01G228700	96.42	41.49	Chalcone synthase
Cytochrome P450 CYP2 subfamily			
Glyma.02G078800	9.07	3.02	Beta-carotene 15,15ʹ-monooxygenase
**Starch and sucrose metabolism**			
Glyma.20G029100	9.2	1.91	Beta-fructofuranosidase
Glyma.11G253900	194.23	78.83	Xyloglucan: endotransglucosylase
Glyma.07G174800	41.02	5.77	Xyloglucan: endotransglucosylase
Glyma.13G322500	48.78	20.69	Xyloglucan:endotransglucosylase
**Nitrogen metabolism**			
Glyma.08G296000	11.21	5.38	Nitrate transporter (NTL1)

**Table 5.** FPKMs and functional categories of genes significantly down-regulated in V1 at S2

Gene name	V1S2	V2S2	Description
**Transcription factors**			
Glyma.03G018000	2.26	7.35	Zinc finger C-x8-C-x5-C-x3-H type
Glyma.12G168600	37.58	98.09	ZF-HD protein dimerization region
**Plant hormone signal transduction**			
Glyma.17G165500	0.38	13.69	Auxin-responsive GH3 gene family
Glyma.11G076200	1.91	6.88	SAUR family protein
Glyma.13G039300	1025.23	2711.05	Gibberellin-regulated protein
Glyma.18G001200	10.85	48.75	Gibberellin-regulated protein
Glyma.09G238300	1101.93	2704.52	Gibberellin-regulated protein
Glyma.13G039600	532.18	1260.11	Gibberellin-regulated protein
**Starch and sucrose metabolism**			
Glyma.10G128200	2.56	7.72	Pyrophosphate-fructose-6-phosphate 1-phosphotransferase
Glyma.07G124100	9.57	22.13	Polygalacturonase
Glyma.20G029300	0.43	8.07	Glycosyl hydrolase family 32
Glyma.06G004400	120.56	252.97	Glycosyl hydrolase family 10
**Flavonoid biosynthesis**			
Glyma.14G072800	0.12	7.21	Bifunctional dihydroflavonol 4-reductase/flavanone 4-reductase
Glyma.09G127700	1.19	7.57	Flavonol 7-O-beta-glucosyltransferase
Glyma.11G000500	6.09	13.46	Flavonol 3-O-glucosyltransferase
**Seed storage**			
Glyma.14G032800	224.24	641.59	Seed storage/LTP family
Glyma.20G248700	251.1	635.31	Seed storage/LTP family
Glyma.09G055100	62.1	158.69	Seed storage/LTP family

In seed coat samples harvested at the S3 stage, 310 and 222 genes were found to be significantly up- or down-regulated, respectively, in the V1 genotype compared with their expression in V2 (Fig. 3A). The up- or down-regulated DEGs are shown in Tables 6 and 7, respectively. Highly expressed genes include genes encoding a TGACG motif-binding factor 4, G-box binding factor 3, MYB domain protein 116, and a cohort of hormone signal transduction genes encoding indole-3-acetic acid 6, BRI1-associated receptor kinase, and an ethylene-forming enzyme. Ubiquitin-mediated proteolysis has been reported to regulate seed size (Li and Li, 2014; Orozco-Arroyo *et al.*, 2015). In our study, a number of ubiquitin-mediated proteolysis candidate genes were found to be significantly enriched in seed coat at the early seed maturation stage (S3-1) in V1. Cytochrome P450 family genes have been reported to positively regulate seed yield in rice and soybean (Wang *et al.*, 2015; Yang *et al.*, 2013). In our study, several candidate cytochrome P450 family genes exhibited high expression levels in the seed coat of the large seed cultivar, V1. Interestingly, a glutathione peroxidase gene was specifically expressed in V1S3-1, but not in the other samples of V1 or any sample of V2. Two leucine-rich repeat protein kinase genes showed over four-fold increased expression in V1S3-1 in comparison with V2S3-1.

**Table 6.** FPKMs and functional categories of genes significantly up-regulated in V1 at S3-1

Gene name	V1S3-1	V2S3-1	Description
**Transcription factors**			
Glyma.08G140100	5.43	0.55	TGACG motif-binding factor 4
Glyma.01G177400	6.99	0.61	G-box binding factor 3
Glyma.09G235100	5.02	0.01	Myb domain protein 116
**Plant hormone signal transduction**			
Glyma.15G012800	11.57	1.39	Indole-3-acetic acid 6
Glyma.11G180700	7.55	0.63	BRI1-associated receptor kinase
Glyma.14G049500	9.12	1.73	Ethylene-forming enzyme
**Ubiquitin-mediated proteolysis**			
Glyma.17G202700	130.87	31.42	CHY-type/CTCHY-type/RING-type Zinc finger protein
Glyma.16G142700	9.65	0.74	LIM domain-containing protein
Glyma.13G136900	14.26	5.01	Ubiquitin-protein ligase 4
Glyma.07G196500	18.65	2.63	Phosphate 2
**Cell wall modification**			
Glyma.16G014100	82.24	14.27	Pectin methylesterase 2
Glyma.12G080100	15.7	4	Xyloglucan endotransglucosylase
Glyma.10G140200	67.16	22.55	Expansin A6
Glyma.02G076400	23.96	3.18	LACCASE
**Starch and sucrose metabolism**			
Glyma.07G237300	80.94	28.25	Plant invertase/pectin methylesterase inhibitor
Glyma.06G314200	144.98	14.99	Plant invertase/pectin methylesterase inhibitor
Glyma.16G107500	3627.02	322.19	Pectin acetylesterase family protein
Glyma.06G179200	76.75	17.6	Galactinol-sucrose galactosyltransferase
Glyma.06G179200	7.28	1.31	Galactinol-sucrose galactosyltransferase
Glyma.16G154600	6.11	0.81	Alpha-1,4-fucosyltransferase
**Cytochrome P450**			
Glyma.01G181900	42.49	3.56	Cytochrome P450 CYP2 subfamily
Glyma.18G080600	5.99	0.45	Cytochrome P450 CYP2 subfamily
Glyma.11G212900	50.53	0.06	Glutathione peroxidase
Glyma.18G043700	5.95	0.05	Glutathione peroxidase
Glyma.15G252200	15.48	1.32	Glutathione S-transferase TAU 19
Glyma.15G252000	86.54	11	Glutathione S-transferase TAU 22
**Protein kinase**			
Glyma.03G177600	35.35	8.43	Leucine-rich repeat protein kinase family protein
Glyma.17G218500	11.15	2.82	Leucine-rich repeat protein kinase family protein

**Table 7.** FPKMs and functional categories of genes significantly down-regulated in V1 at S3-1

Gene name	V1S3-1	V2S3-1	Description
**Transcription factors**			
Glyma.02G109800	1.74	15.73	NAC-like, activated by AP3/PI
Glyma.10G272300	3.54	16.78	Zinc finger protein with KRAB and SCAN domains
Glyma.18G061800	3.81	31.87	Jasmonate-inducible protein-related
**Carbohydrate metabolic process**			
Glyma.11G095100	2.81	101.73	Glycosyl hydrolase family 17
Glyma.12G088100	15.26	55.02	Glycosyltransferase family 29 (sialyltransferase) family protein
**Flavonoid biosynthesis**			
Glyma.08G220200	8.09	23.01	Hydroxycinnamoyl-CoA shikimate/quinate hydroxycinnamoyl transferase
Glyma.02G136100	3.57	18.54	Flavanone 3-dioxygenase.
**Transporter activity**			
Glyma.01G174300	0.02	9.61	ABC transporter family protein
Glyma.06G248800	11.28	29.78	ABC-2 transporter family protein
Glyma.19G011600	1.03	5.36	EamA-like transporter family protein MtN21
**Cell wall biogenesis**			
Glyma.03G227600	1.65	8.2	Pectinacetylesterase family protein
Glyma.19G145200	1.36	14.82	Polygalacturonase inhibiting protein 2

In the S3 cotyledon samples, 241 genes were significantly differentially expressed in pairwise comparisons between V1S3-2 and V2S3-2, with 152 genes up- and 89 genes down-regulated in V1S3-2 relative to V2S3-2 (Fig. 3A). Genes encoding some transcription factors—a helix-loop-helix DNA-binding domain, nuclear factor Y subunit B12, NAC domain-containing protein 6 and auxin-responsive proteins—exhibited significantly higher expression in V1 cotyledons compared with V2 cotyledons. A WRKY transcription factor family protein and a heat shock transcription factor A1E each displayed over two-fold higher expression in V1S3 cotyledons compared with V2S3 cotyledons. ACC oxidase 1 was significantly enriched in V1 at stage S3-2. Soybean CYP78A72, an ortholog of *KLU* which positively regulates seed size in soybean (Zhao *et al.*, 2016), was significantly enriched in the cotyledon of V1 but not in V2. Functional categories for the up- or down-regulated DEGs are shown in Tables 8 and 9.

**Table 8.** FPKMs and functional categories of genes significantly up-regulated in V1 at S3-2

Gene name	V1S3-2	V2S3-2	Description
**Transcription factors**			
Glyma.03G176600	14.07	5.59	WRKY family transcription factor family protein
Glyma.05G036800	23.61	1.97	Helix-loop-helix DNA-binding domain
Glyma.04G052000	46.17	5.85	Heat shock transcription factor A1E
Glyma.18G122900	54.09	0.36	Nuclear factor Y subunit B12
Glyma.18G261300	11.97	0.01	NAC domain containing protein 6
Glyma.03G014800	5.31	0.15	Auxin-responsive protein
**Ethylene biosynthetic process**			
Glyma.05G222400	52.93	4.12	ACC oxidase 1
**Carbohydrate metabolic process/plant cell wall modification**			
Glyma.13G215500	6.58	0.5	Callose synthase 5
Glyma.20G082700	9.1	0.07	Xyloglucan endotransglucosylase
Glyma.06G200200	12.71	0.01	Xyloglucan endotransglucosylase
Glyma.06G200800	8.66	0.01	Xyloglucan endotransglucosylase
Glyma.04G201600	17.45	0.22	Glycosyl hydrolase
Glyma.19G066300	17.8	0.16	Xyloglucan endotransglucosylase
Glyma.U004500	6.02	0.02	Glycoside hydrolase starch-binding domain-containing protein
Glyma.02G119600	635.92	220.25	Cytochrome P450 CYP2 subfamily
**Fatty acid degradation**			
Glyma.14G121200	87.61	4.69	Alcohol dehydrogenase 1

**Table 9.** FPKMs and functional categories of genes significantly down-regulated in V1 at S3-2

Gene name	V1S3-2	V2S3-2	Description
**MAPK signaling**			
Glyma.02G076600	152.85	2731.19	HSP20 family protein
**Storage protein related**			
Glyma.13G194400	3.52	23347	Albumin I
Glyma.19G236600	29.84	1854.67	Basic 7S globulin-related
**Others**			
Glyma.10G027600	13.89	74.51	Seed maturation protein
Glyma.16G049000	13.43	143.95	Light-regulated protein Lir1
Glyma.15G154500	0.06	9.11	MTERF
Glyma.19G004800	14.35	73.08	Oleosin
Glyma.13G224400	0.01	65.3	Other RNA
Glyma.05G149700	5.63	39.72	Phragmoplast-associated kinesin-related protein, putative
Glyma.08G280100	0.01	9.11	Transducin family protein/WD-40 repeat family protein
Glyma.09G246000	0.15	10.47	UDP-glucosyltransferase 85A3
Glyma.14G077100	0.8	7.13	Uncharacterized conserved protein
Glyma.10G122800	0.01	7.08	Unknown protein
Glyma.09G234700	0.04	16.23	Unknown protein
Glyma.10G170100	0.36	44.91	Unknown protein
Glyma.11G119000	0.33	9.84	Unknown protein

### Identification of DEGs among different seed developmental stages

**Fig. 4.**
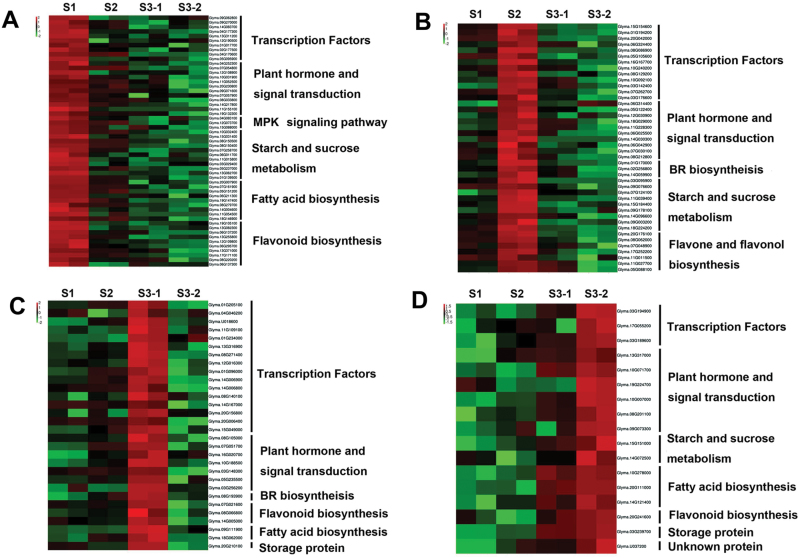
**Heatmap comparison of the DEGs at each seed developmental stage.** Functional categories of significantly over-represented DEGs in S1 (A), S2 (B), S3-1 (C), and S3-2 (D). (This figure is available in colour at *JXB* online.)

Pairwise comparisons were also performed to identify the DEGs among the three developmental stages in both genotypes. The numbers of DEGs for S1, S2, S3-1 and S3-2 are shown in Fig. 3B. Genes significantly differentially expressed in stage S1 were revealed by pairwise comparisons with the S2, S3-1, and S3-2 stages (Supplementary Table S4). Functional categories for the S1-enriched DEGs are shown in Fig. 4A. A large number of transcription factors and hormonal signal transduction genes were found to be highly enriched in S1 in both V1 and V2. A cell division marker gene *cyclin D3*, *Glyma.11G052500*, was highly expressed in S1 in both varieties, indicating its function in cell division during the seed set stage. Multiple plant hormone signal transduction genes were also highly enriched in S1. The analyses further identified several genes involved in metabolism in the S1 stage. These include genes encoding proteins for starch and sucrose metabolism, fatty acid biosynthesis, and flavonol biosynthesis (Fig. 4A). These data indicate that a regulatory network involving hormone signaling and sugar, flavonoid, and fatty acid metabolism governs seed set in soybean.

Similar analyses identified genes significantly differentially expressed in S2 stage relative to expression in S1, S3-1, and S3-2 samples (Fig. 4B, Supplementary Table S5). Functional categories for the S2-enriched DEGs are shown in Fig. 4B. Here, 10 transcription factors were highly enriched in developing seeds at the S2 stage. Three cytochrome P450 family 90 genes, *Glyma.01G170000*, *Glyma.02G256800*, and *Glyma.14G059900* involved in brassinosteroid (BR) biosynthesis, were significantly enriched in the S2 stage. Interestingly, an invertase inhibitor gene, *Glyma.09G076600*, was induced in S2. Invertase inhibitors suppress invertase activity, thus blocking the hydrolysis of sucrose to glucose and fructose (Ruan, 2014). Increased invertase activity can promote cell division for seed set (Ruan *et al.*, 2012). The expression of the invertase inhibitor could help to slow down cell division and promote cell expansion, characteristic of seed development at the S2 stage. Some flavone and flavonol biosynthesis genes were also enriched in S2.

Genes significantly differentially expressed in seed coat of S3 stage (S3-1) are shown in Supplementary Table S6, and their functional categories are shown in Fig. 4C. Elevated transcript levels were identified in stage 3-2 for 15 transcription factor-encoding genes, belonging to the basic-leucine zipper (bZIP) family, GATA-type zinc finger family, GRAS family, WRKY family, squamosa promoter-binding protein-like (SBP domain) family, and a heat shock transcription factor. Multiple plant hormone signal transduction genes, including those encoding an abscisic acid (ABA)-responsive element binding factor, cytokine receptor AHK2/3/4, an ethylene receptor, a SAUR family protein, a gibberellin receptor GID1, a jasmonate ZIM domain-containing protein and a jasmonic acid-aminosynthetase, and a cytochrome P450D90 which is part of the BR biosynthesis pathway, were also significantly enriched in S3-1 samples (Supplementary Table S6).

Genes significantly differentially expressed in seed cotyledons of the S3 stage (S3-2) are shown in Supplementary Table S7. Functional categories for the S3-2 enriched DEGs are shown in Fig. 4D. Genes encoding a bZIP and a GATA-type zinc finger transcription factor were enriched in the S3 cotyledons. Other genes significantly enriched in the S3-2 stage include those involved in multiple plant hormone signal transduction pathways, such as an ABA-responsive element binding factor, a phytochrome-interacting factor 3, an ethylene-responsive transcription factor AP2/ERF 59, a histidine-containing phosphotransfer protein, and a SAUR family protein, as well as flavonoid and unsaturated fatty acid biosynthesis genes. Transcripts involved in the starch and sucrose metabolic pathway and storage protein synthesis were also significantly enriched in the S3-2 stage.

### Construction of gene coexpression networks

**Fig. 5.**
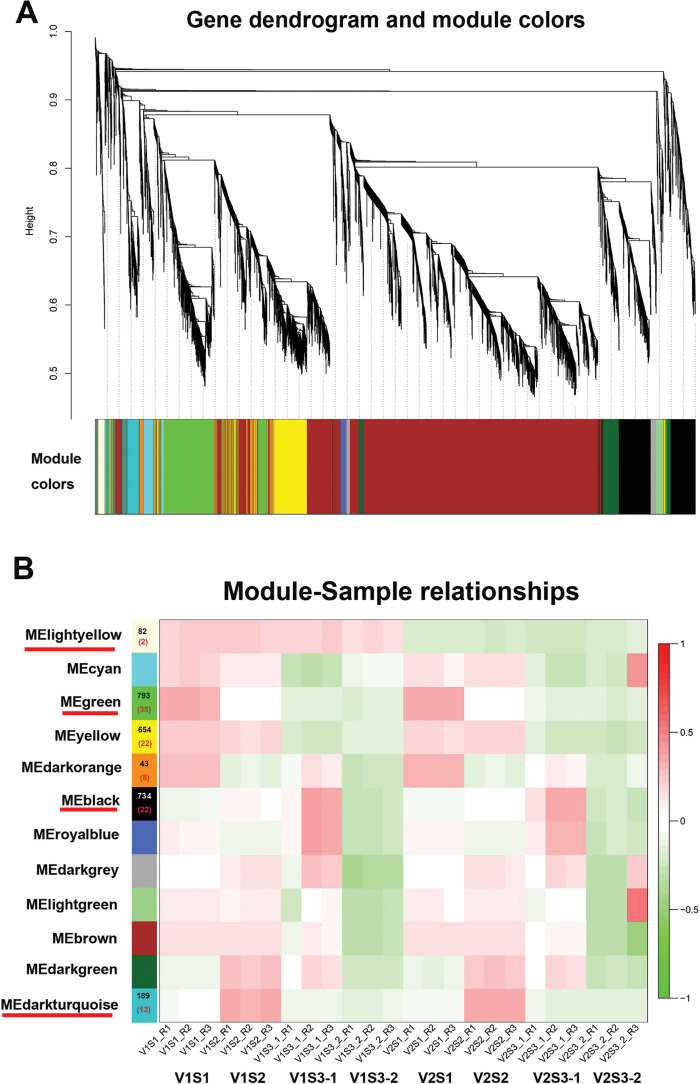
**WGCNA of genes in V1 and V2 at each seed developmental stage.** (A) Hierarchical cluster tree showing coexpression modules identified by WGCNA. Each leaf in the tree represents one gene. The major tree branches constitute 12 modules, labeled with different colors. (B) Module–sample association. Each row corresponds to a module, labeled with a color as in (A). The number of genes in each module is indicated on the left. The number of transcription factors in each module is indicated by the number in parentheses. Each column corresponds to a specific tissue and replicate (R). The color of each cell at the row–--column intersection indicates the correlation coefficient between the module and the tissue type. A high degree of correlation between a specific module and the tissue type is indicated by red underline of the module name. (This figure is available in colour at *JXB* online.)

To obtain a comprehensive understanding of genes expressed in the successive developmental stages across the two genotypes, and to identify the specific genes that are highly associated with seed size, a WGCNA was performed (Langfelder and Horvath, 2008). After filtering out the genes with a low expression (FPKM <0.05), 7359 genes were retained for the WGCNA. Coexpression networks were constructed on the basis of pairwise correlations of gene expression across all samples. Modules were defined as clusters of highly interconnected genes, and genes within the same cluster have high correlation coefficients among them. This analysis identified 12 distinct modules (labeled with different colors) shown in the dendrogram in Fig. 5A, in which major tree branches define the modules. The 12 modules correlated with distinct samples due to sample-specific expression profiles (Supplementary Table S8). Notably, 4 out of 12 coexpression modules are composed of genes that were highly expressed in a single sample type; these are indicated with red underlines in Fig. 5B.

The lightyellow module identified 82 genes specific to the large-seed (V1) phenotype across all the developmental stages (Supplementary Table S9). The green module, with 793 identified genes, was highly associated with S1 (Supplementary Table S10). The darkturquoise module (189 genes) was highly associated with S2 (Supplementary Table S11). The black module, representing 734 genes, was highly associated with S3-1 (Supplementary Table S12). The yellow module, containing 654 genes, was highly related to the S1 and S2 stages (Supplementary Table S13).

WGCNA can also be employed to construct gene networks, in which each node represents a gene and the connecting lines (edges) between genes represent coexpression correlations (Langfelder and Horvath, 2008). Hub genes are those that show most connections in the network as indicated by their high K_ME_ (eigengene connectivity) value. The top six genes with the highest K_ME_ values in each of the specific modules are shown in Table 10.

**Table 10.** Candidate hub genes in S1, S2, S3-1, and V1 modules

Gene name	Description	K_ME_ value
**S1-specific green module**		
Glyma.07G146800.1	Glycerol-3-phosphate acyltransferase	0.99
Glyma.15G140200.1	ATP citrate (pro-S)-lyase	0.99
Glyma.08G150500.1	Beta-glucosidase	0.99
Glyma.02G132200.1	Abscisic acid 8ʹ-hydroxylase	0.99
Glyma.10G202400.1	Beta-carotene 15,15ʹ-monooxygenase	0.99
Glyma.20G007900.1	Long-chain acyl-CoA synthetase	0.99
**S2-specific darkturquoise module**		
Glyma.18G297300.1	Adenylate isopentenyltransferase (cytokinin synthase)	0.99
Glyma.16G066600.1	Legumain	0.99
Glyma.09G048400.1	Peroxidase	0.99
Glyma.13G046400.1	Cytokinin trans-hydroxylase	0.99
Glyma.01G170000.1	Cytochrome P450, family 90, subfamily C, polypeptide 1	0.99
Glyma.06G101700.1	3-deoxy-7-phosphoheptulonate synthase	0.99
**S3-1-specific black module**		
Glyma.17G083400.1	Agmatine deiminase	0.99
Glyma.10G152200.1	Respiratory burst oxidase	0.99
Glyma.10G208300.2	Rab family, other	0.98
Glyma.07G060900.1	Translation initiation factor eIF-1A	0.98
Glyma.06G077300.1	Cyclic nucleotide gated channel, other eukaryote	0.98
Glyma.08G157400.1	Callose synthase	0.98
Glyma.04G034200.1	Leishmanolysin	0.87
Glyma.14G002400.1	BR-signaling kinase	0.86
**V1-specific lightyellow module**		
Glyma.05G130300.2	DNA-directed RNA polymerase II subunit RPB7	0.99
Glyma.08G210900.3	Peptide deformylase	0.98
Glyma.10G175400.1	Translation initiation factor eIF-6	0.97
Glyma.16G101900.1	Capping protein (actin filament) muscle Z-line, beta	0.96
Glyma.05G130300.4	DNA-directed RNA polymerase II subunit RPB7	0.96
Glyma.13G190900.4	Mitochondrial inner membrane protease subunit 1	0.95

**Fig. 6.**
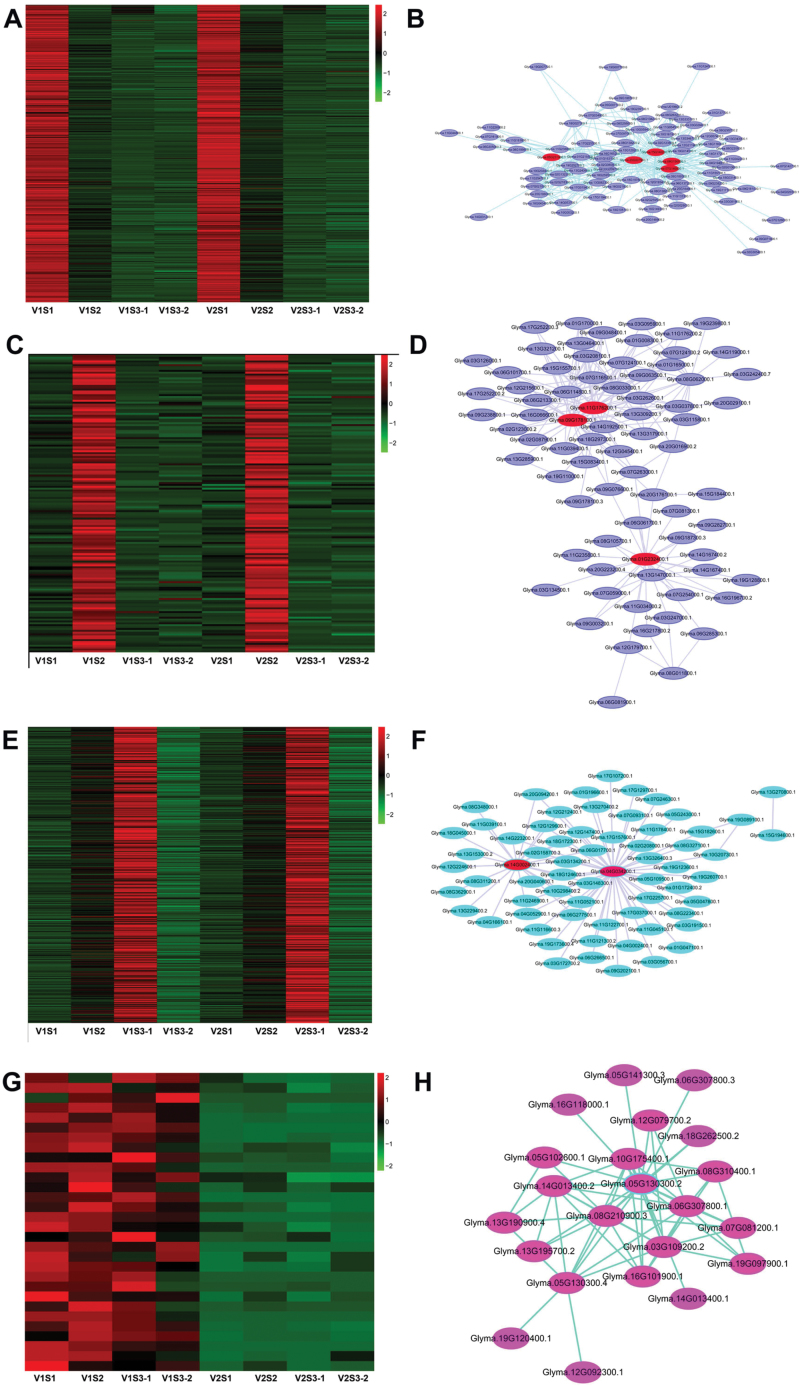
**Coexpression network analysis of stage-specific modules.** (A), (C), (E), and (G) Heatmaps showing genes in each module that were significantly over-represented in S1, S2, S3, and V1, respectively. (B), (D), (F), and (H) The correlation networks in the module corresponding to (A), (C), (E), and (G), respectively. Candidate hub genes are shown in red. (This figure is available in colour at *JXB* online.)

Heatmaps (Fig. 6A) show that the green module-specific genes were over-represented in S1. In the green module, 35 S1-specific genes encode transcription factors, including several MYB family transcription factors, a homeobox-leucine zipper protein, an EREBP-like factor, and an AP2-like factor (ANT).The correlation network of the green module is shown in Fig. 6B. Glycerol-3-phosphate acyltransferase, ATP citrate (pro-S)-lyase, beta-glucosidase, abscisic acid 8ʹ-hydroxylase, beta-carotene 15, 15ʹ-monooxygenase, and long-chain acyl-CoA synthetase were identified as candidate hub genes for this module (Fig. 6B, Table 10).

The darkturquoise module genes were over-represented in S2 (Fig. 6C). In the S2-specific module, 13 genes encode transcription factors, including ABA-responsive element-binding factor, a MADS-box transcription factor, an AP2-like factor (ANT), and a homeobox-leucine zipper protein. The correlation network of the darkturquoise module is shown in (Fig. 6D). Genes encoding cytokinin synthase, legumain, peroxidase, cytokinin trans-hydroxylase, cytochrome P450, family 90, and 3-deoxy-7-phosphoheptulonate synthase were identified as candidate hub genes for this module (Fig. 6D, Table 10).

The black module genes were over-represented in S3-1 (Fig. 6E). These included 21 of 734 genes encoding ABA-responsive element-binding factor, plant G-box-binding factor, transcription factor TGA, EREBP-like factor, MADS-box transcription factor, AP2-like factor (ANT), and a homeobox-leucine zipper protein. The correlation network of the black module is shown in Fig. 6F. A BR-signaling kinase gene was identified as a candidate hub gene for this module (Fig. 6F, Table 10). Several BR biosynthesis genes were also enriched in the seed coat, indicating that a BR signaling regulatory network may play a major role in seed coat development.

The lightyellow module genes were more highly expressed in V1 (Fig. 6G). Two of the 82 V1-specific genes encode transcription factors, homeobox-leucine zipper protein and EREBP-like factor. DNA-directed RNA polymerase II subunit RPB7 was identified as the candidate hub gene for this module (Fig. 6H, Table 10).

### Expression of seed size marker gene in the RNA-Seq data

The Arabidopsis genes *KLU*, *WRKY44*, and *STK* have been identified genetically as regulators of seed size (Adamski *et al.*, 2009; Garcia *et al.*, 2005; Mizzotti *et al.*, 2012; Zhao *et al.*, 2016). The expression of the soybean homologues of these genes in the RNA-Seq data is shown in Supplementary Fig. S4. The soybean *KLU* and *WRKY44* genes showed significant increases in transcript level in seed cotyledon (S3-2) of the V1 seeds (*P*<0.05, Student’s *t*-test) compared with the small seed genotype V2 (Supplementary Fig. S4A). *WRKY44* exhibited significant increases in transcript level in the S2, S3-1, and S3-2 developmental stages of large-seed V1 (*P*<0.05, Student’s *t*-test) compared with V2 (Supplementary Fig. S4B). *STK* showed a significant increase in transcript abundance in the S3-1 stage of V1 (*P*<0.05, Student’s *t*-test) (Supplementary Fig. S4C).

### Overexpression of a cytochrome P450 family gene (*CYP78A5*) increased seed size and seed weight

**Fig. 7.**
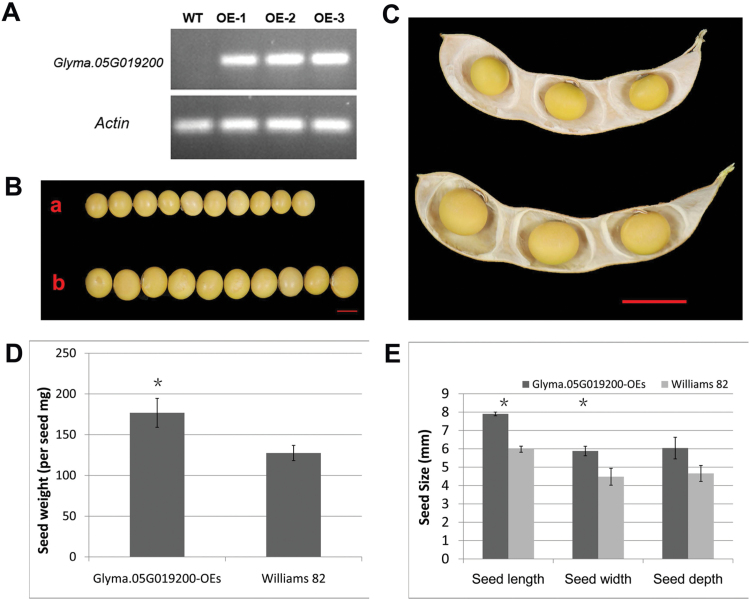
**Overexpression of *GmCYP78A5* (*Glyma.05G019200*) increased seed size in transgenic soybean.** (A) RT-PCR analysis of the expression level of *Glyma.05G019200 in* Williams 82 and three *Glyma.05G019200* overexpression transgenic lines. RNA was extracted from developing seeds at the S3 stage. (B) Seeds and (C) pods of *Glyma.05G019200* overexpression transgenic lines and their non-transgenic segregants. (D) Seed weight and (E) seed dimensions of *Glyma.05G019200* overexpression transgenic lines and their non-transgenic segregants. Values are reported as means±SE (*n*=3). * Significant difference (*P<*0.05, Student’s t-test). (This figure is available in colour at *JXB* online.)

A cytochrome P450 family gene, *GmCYP78A5* (*Glyma.05G019200*), was found to be involved in soybean seed size and pod number by association analysis (Wang *et al.*, 2015). This gene was more highly expressed in V1S2 samples compared with V2S2 (Supplementary Fig. S5). To assess the role of *GmCYP78A5* in soybean seed size determination, *GmCYP78A5* was overexpressed in the soybean variety Williams 82 under the seed-specific promoter of the soybean β-conglycinin α subunit encoding gene (*Glyma.20G148300.1*) (Yoshino *et al.*, 2006). Three transgenic lines with significantly higher expression levels of *GmCYP78A5* in developing seeds of the T1 generation were selected for further analysis (Fig. 7A). The high expression of the transgene in the developing seeds confirmed the success of transformation and the suitability of the seed-specific promoter from β-conglycinin α subunit. The seed size and weight of T1 transgenic seeds were significantly increased in these transgenic lines compared with respective values in the wild-type plants (Fig. 7B, C, D, E).

## Discussion

Understanding the molecular mechanisms controlling seed set and size is of great importance for improving seed yield in many crop species, including soybean. In this study we performed transcriptomic comparison between a large-seed and a small-seed genotype of soybean across the early seed developmental stages. The results provide comprehensive information on genes involved in the determination of seed size. This study also shows that WGNCA is particularly useful in identifying sample-specific modules and candidate hub genes.

### Soybean seed yield is largely determined at an early seed developmental stage

The seed size of the V1 and V2 genotypes exhibited a significant difference as early as the seed set stage, S1 (Fig. 1A), suggesting that genes and networks controlling soybean seed size function at this early stage (Tables 2 and 3) and, to a lesser extent, at the S2 stage (Tables 4 and 5). The differences in gene expression between the V1 and V2 genotypes in the S3-1 and S3-2 stages may mostly be the consequence of the differential expression of genes at S1 and S2.

Genes encoding four protein kinases, including the CBL-interacting protein kinases 13 and 23 and two leucine-rich repeat transmembrane protein kinase family proteins, were significantly up-regulated in the large seed V1S1 sample relative to their expression in V2S1 (Table 2). Transmembrane receptor-like kinases and receptor-like cytoplasmic kinases have been reported to regulate hormone signaling, seed development, and seed size (Luo *et al.*, 2005; Yu *et al.*, 2014). The high expression levels of the above kinase-encoding genes specific for the S1 stage imply that they have a role in large seed size determination at the seed set stage. Flavonoids, a group of plant secondary polyphenolic metabolites, have been found to regulate seed size by communicating growth signals between seed coat and the endosperm (Doughty *et al.*, 2014). Consistently, two genes encoding a flavonol 3-O-glucosyltransferase and a flavonol 7-O-beta-glucosyltransferase were highly expressed in V1S1 in the current study, suggesting a potential role of these secondary metabolism genes in modulating soybean seed size.

R2R3 MYB and MYB56 transcription factors have been found to regulate seed size and shape in Arabidopsis (Zhang *et al.*, 2013). A gene encoding a MYB-like DNA-binding protein (*Glyma*.*12G100600*) displayed specific and significantly increased mRNA levels in V1 compared with levels in V2 at S2 (Table 4), indicating the involvement of this DNA-binding protein in controlling soybean seed size. Transcripts of three genes encoding xyloglucan endotransglucosylase were significantly enriched in V1S2, suggesting that these genes may play roles in cell wall formation during cell expansion at the seed growth stage, S2.

### Molecular elements in the seed coat and embryo likely contribute to the difference in soybean seed size

A large number of genes were differentially expressed between V1 and V2 in in the seed coat of stage S3 seeds (Tables 6 and 7). These genes may contribute to maintaining or enhancing the size difference initiated at the S1 and S2 stages. Previous studies showed that ubiquitin-mediated proteolysis was found to be involved in seed size determination (Du *et al.*, 2014; Li and Li, 2014; Li *et al.*, 2008; Xia *et al.*, 2013). In our study, an E3 ligase RING-type Zinc finger protein (*Glyma.17G202700*) was highly enriched in the seed coat of V1. In addition, the expression of the E2 conjugase phosphate 2 (*Glyma.07G196500*) was seven times higher in the seed coat of V1 relative to V2. This result suggests that an ubiquitin-mediated proteolysis pathway operating in the seed coat may play a role in seed size determination.

Interestingly, some cell wall modification genes were highly expressed at stage S3-1 in V1 (Table 6). The transcript levels of several genes controlling starch and sucrose metabolism, notably, two invertase inhibitor genes and a pectin acetylesterase gene, were also increased in V1. An invertase inhibitor can suppress invertase activity, that is, the hydrolysis of sucrose to glucose and fructose. Invertase-derived glucose has been shown to promote cell division, probably through sugar signaling (Ruan, 2014). The S3 stage is characterized by the seed filling process (Supplementary Table S1). Repressed invertase activity by high expression of invertase inhibitor genes could lead to a low level of glucose signaling, thereby suppressing or terminating cell division and promoting the seed filling process.

Of significance to note is the strong expression of a group of genes encoding a cytochrome P450 in the *CYP2* subfamily, a glutathione peroxidase, and a glutathione S-transferase, TAU 22, in the seed coat of V1. Cytochrome P450 family genes are involved in a variety of biochemical pathways for the production of a range of metabolites and hormones (Schuler and Werck-Reichhart, 2003). Cytochrome P450 family members have been recognized to regulate organ size and development. For example, overexpression of *CYP78A6* significantly increased seed size in Arabidopsis, while *CYP78A6* deletion mutants exhibited a small seed phenotype (Fang *et al.*, 2012). We found that mRNA of a soybean ortholog of the cytochrome P450 family gene *KLU* (*Glyma.02G119600*) was enriched in seed coat at the S3 stage in the V1 (large seed) genotype. Its close homolog, *Glyma.05G019200* (*GmCYP78A10*), was positively associated with soybean seed size and pod number (Wang *et al.*, 2015). Indeed, overexpression of *Glyma.05G019200* enhanced soybean seed size (Fig. 7), confirming its role in seed size determination.

In parallel to molecular events in the seed coat, those in the embryo are equally important to seed development and the determination of seed size. Here, a gene encoding a nuclear factor Y subunit B12 was significantly enriched in S3-2 of V1. Nuclear factor Y family proteins are involved in protein and oil accumulation (Lu *et al.*, 2016). Interestingly, a gene encoding a helix-loop-helix DNA-binding domain protein was also highly expressed in the large seed cotyledons at stage S3-2 of V1. Its function in seed development is yet to be determined.

### Stage-specific or large seed-specific modules and hub genes were identified by using WGNCA

Another novel finding from this study is that, by performing WGNCA, we identified seed developmental stage-specific or seed size-specific gene modules (Fig. 6A, C, E, G, Table 10). To this end, four AP2-like factor genes were identified as S1 stage-specific modules, comprising genes encoding an ANT lineage family protein, two MADS-box transcription factors, and a homeobox-leucine zipper protein. AINTEGUMENTA (ANT) is a transcription factor that belongs to the AP2-like family (Elliott *et al.*, 1996; Klucher *et al.*, 1996). Moderate overexpression of the *ANT* gene in Arabidopsis resulted in a larger embryo (Mizukami and Fischer, 2000). MADS-box proteins define a fairly large group of transcription factors that are involved in Arabidopsis flower and fruit development (Fan *et al.*, 2013). Several MADS-box proteins have also been reported to regulate endosperm development (Kang *et al.*, 2008). A total of 752 S1 module genes were enriched in the photosynthesis, carbon fixation, and cell cycle pathways (Supplementary Table S10); these genes are clearly important for active cell division and utilization of assimilates in the newly formed seed.

Cytokinin and BR pathway genes were among the highly expressed S2-specific module genes. These include several histidine-containing phosphotransfer protein genes and BR biosynthesis and signal transduction genes. Importantly, a cytokinin trans-hydroxylase gene was identified as a candidate hub gene for the S2 module. Studies in Arabidopsis and maize indicate that cytokinin signaling has a predominant role in early endosperm development (Day *et al.*, 2008). The main role of cytokinins in seed development is to modulate cell division in the endosperm (Riou-Khamlichi *et al.*, 1999). The BR biosynthesis/signaling genes have been reported to regulate seed development in rice. For example, mutation in these genes reduced seed length (Hong *et al.*, 2005; Morinaka *et al.*, 2006; Tanabe *et al.*, 2005). There is also evidence in rice that BRs may regulate seed size by stimulating the flow of assimilates from source to sink tissues in the grain filling stage (Wu *et al.*, 2008). The S2 stage is characterized by active cell expansion and storage product synthesis. Our identification of these stage-specific plant hormone biosynthesis and signal transduction genes as candidate regulators for soybean seed filling provide molecular targets for further studies to examine their roles in seed filling.

Also noteworthy is that multiple hormone pathway genes were enriched in seed coat at the S3 stage. These include two BR biosynthesis genes and one gene for BR-signaling kinase, as well as genes encoding a gibberellin 2-oxidase, a gibberellin receptor GID1, a phytochrome-interacting factor 3, an ethylene receptor, an EIN3-binding F-box protein, an ABA-responsive element-binding factor, an Arabidopsis histidine kinase 2/3/4, and an auxin-responsive protein IAA. Moreover, a large number of ubiquitin-mediated proteolysis genes (ubiquitin-conjugating enzyme E2A, E2C, E2W, E2N, E2D/E, E2G1, E2J1, E2J2) were also overrepresented in the S3 seed coat-specific module.

Physiological and genetic studies have suggested that multiple phytohormones are involved in the regulation of seed coat development, including auxins, cytokinins, gibberellins, and brassinolides (Divi and Krishna, 2009; Müller and Sheen, 2007). We identified the above mentioned genes in the seed coat during the middle seed growth stage, indicating that a network for complex hormonal synthesis and signaling is operating in the seed coat to regulate seed development. The BR signaling pathway receptor BRI1-associated receptor kinase is highly expressed in the seed coat and was identified as a candidate hub gene in seed coat at the early maturation stage.

Although the molecular and physiological functions of many stage-specific genes identified in this study are unknown, some are known regulators of seed development in other plant species. These include AP2-like factor (*ANT*) (Elliott *et al.*, 1996; Klucher *et al.*, 1996), TTG2, MADS-box transcription factor (Kang *et al.*, 2008), and *KLU* (Anastasiou *et al.*, 2007). This suggests that the stage-specific genes identified here likely play critical roles in soybean seed development.

Overall, the analyses of the comprehensive transcriptome data set in this study provide a useful genomic resource for the grain legume research community, and molecular insights into the transcriptomic network underpinning seed set and growth in soybean.

## Supplementary data

Supplementary data are available at *JXB* online.

Fig. S1. Comparison of seed length and fresh weight between V1 and V2.

Fig. S2. Map of construct to generate transgenic soybean overexpressing *GmCYP78A5.*

Fig. S3. Experimental validation of the DEGs expression by qRT-PCR.

Fig. S4. Expression of seed size marker genes in each developmental stage of V1 and V2.

Fig. S5. Expression analysis of *Glyma.05G019200*.

Table S1. Ontology of soybean seed developmental stages.

Table S2. List of differentially expressed genes between V1 and V2 in each developmental stage.

Table S3. List of differentially expressed genes in each developmental stage in both of V1 and V2.

Table S4. FPKMs and function categories of genes significantly enriched in S1.

Table S5. FPKMs and function categories of genes significantly enriched in S2.

Table S6. FPKMs and function categories of genes significantly enriched in S3-1.

Table S7. FPKMs and function categories of genes significantly enriched in S3-2.

Table S8. Genes list of 12 modules.

Table S9. Genes list of V1 specific module.

Table S10. Genes list of S1 specific module.

Table S11. Genes list of S2 specific module.

Table S12. Genes list of seed coat (S3-1) specific module.

Table S13. Genes list of S1 S2-specific modules.

Table S14. Primers for RT-PCR.

Table S15.Gene expression level shown in FPKM.

Table S16.Gene expression level shown in TPM.

Table S17. Numbers of reads mapped to genome.

Table S18. Scripts for DEGs and WGNCA.

## Supplementary Material

supplementary_figures_S1_S5

Supplementary_tables_S1_S4_S14_S17_S18

supplementary_table_S2

supplementary_table_S3

supplementary_table_S15

supplementary_table_S16

supplementary_tables_S19
