# Supplementary material for: Identification of regulatory networks and hub genes controlling soybean seed set and size using RNA sequencing analysis
Source: J Exp Bot. 2017 Jan 13;68(8):1955–72. doi: 10.1093/jxb/erw460 (PMC5429000; doi:10.1093/jxb/erw460)
Supplement: supplementary_figures_S1_S5 [file erw460_suppl_supplementary_figures_s1_s5.pdf]

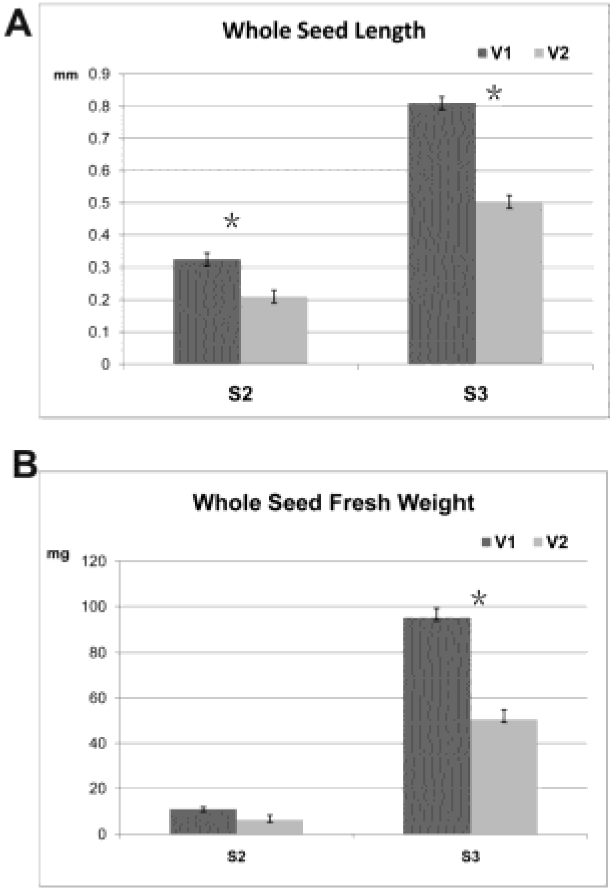

Fig. S1. Comparison of seed length (A) and fresh weight (B) between V1and V2. Statistical analysis of the seed length of whole seed length of S2 and S3 stages between V1 and V2, V1 showed significantly increase in seed length in S2 and S3 stages(Student's *t*-test $P < 0.05$, indicate with＊). Three biological replicates were used for measurement.

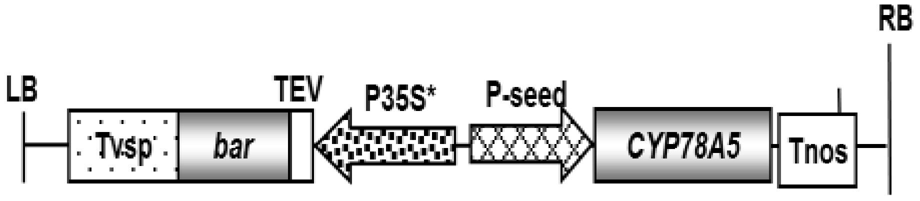

Fig. S2. T-DNA region of constructs for soybean transformation. LB, left border; RB, right border; bar, phospinothricin transferase gene; CYP78A5, *GmCYP78A5 (Glyma.05G019200* gene. P-seed, a soybean seed-specific promoter from β-conglycinin α subunit (Glyma.20G148300.1) (Yoshino et al., 2006).

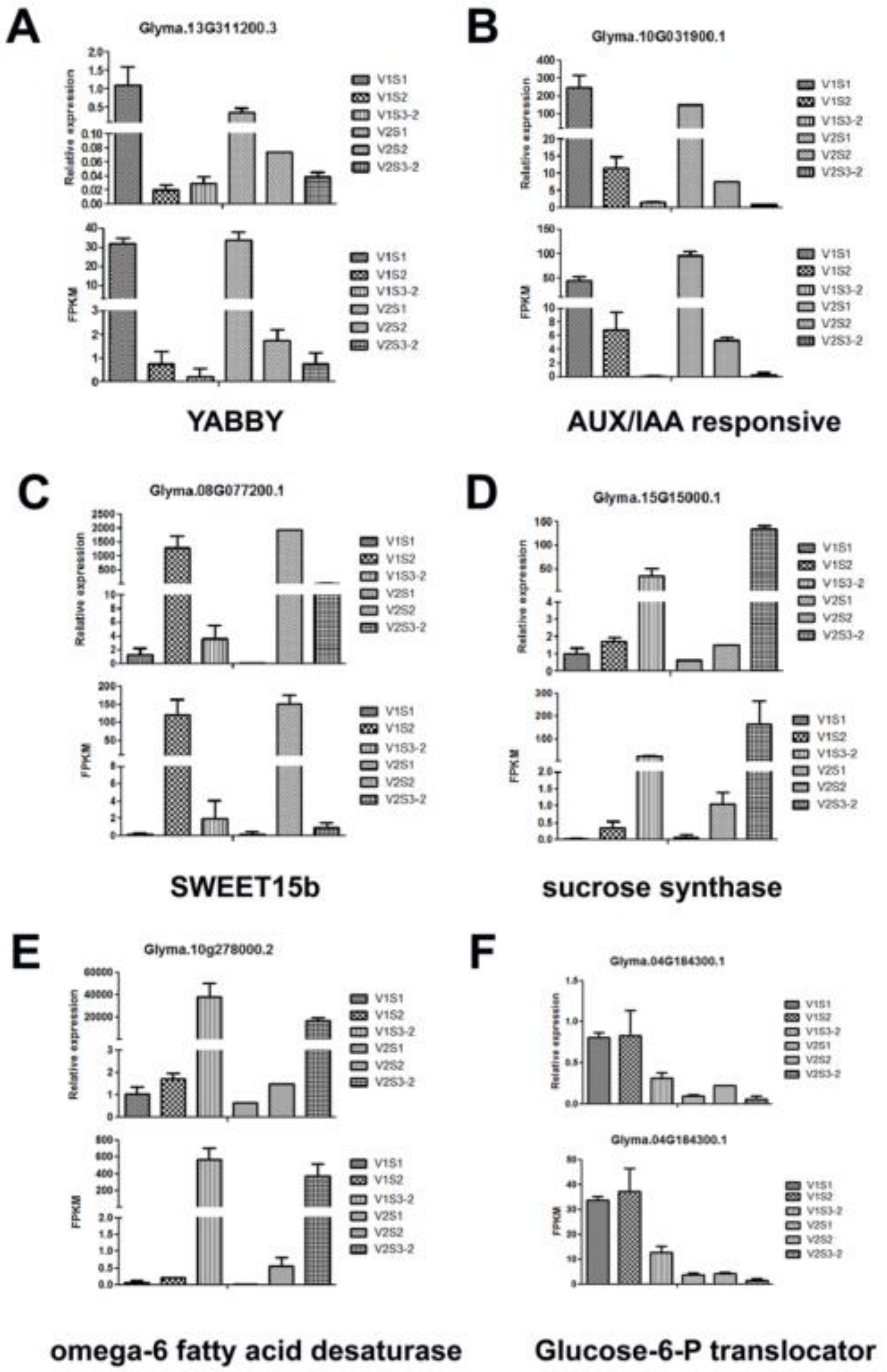

Fig. S3. Experimental validation of the DEGs expression by qRT-PCR. (A)-(F). qRT-PCR analysis of six differential expressedgenes in different samples. Transcriptabundancechanges were detected by FPKMvaluesaccording toRNA-Seq. Values are reported as means±SEs (n = 3). Three biological replications were included in the qRT-PCR analysis.

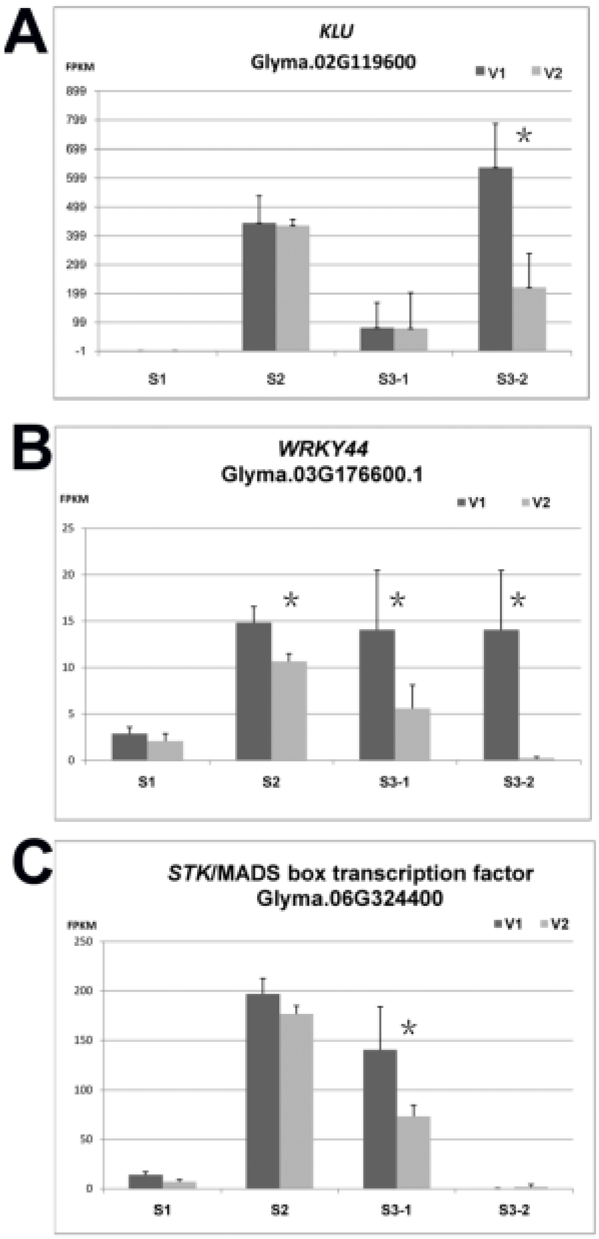

Fig. S4. Expression of seed size marker genes in each development stage of V1 and V2. The transcript abundance of soybean homologues of Arabidopsisseed size marker genes, *GmKLU* (A), *GmWRKY44* (B), *GmSTK* (C), are detected by FPKM value according to RNA-Seq. Values are reported as means±SEs (n = 3). Three biological replicates were included. Symbol * indicate the significant difference (P < 0.05; Student's *t*-test).

A

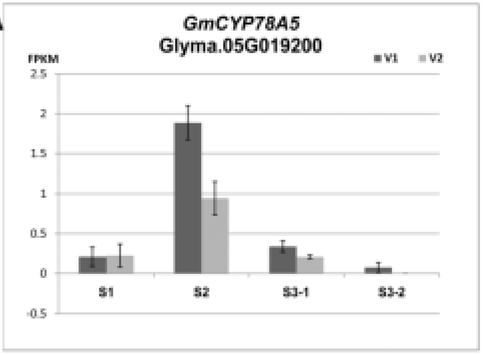

Fig. S5 Expression analysis of *Glyma.05G019200*

A. Expression analysis of *Glyma.05G019200* in each developmental stage of V1 and V2
